# Impact of moderate and extreme climate change scenarios on growth, morphological features, photosynthesis, and fruit production of hot pepper

**DOI:** 10.1002/ece3.3647

**Published:** 2017-11-26

**Authors:** Sang Gyu Lee, Sung Kyeom Kim, Hee Ju Lee, Hee Su Lee, Jin Hyoung Lee

**Affiliations:** ^1^ Vegetable Research Division National Institute of Horticultural & Herbal Science Wanju Korea

**Keywords:** *Capsicum annuum* L. chili, CO_2_ concentration, precipitation, temperature, weather

## Abstract

Horticultural crop production and changes in physiological aspects during the growing season may be affected by climate change factors (CC), which include increased temperature and the associated doubling or tripling of atmospheric CO_2_ concentrations. However, the potential effects are complex and many parameters might impact on the observed effects. To evaluate the effects of CC, the growth, yield, fruit characteristics, photosynthetic traits, and morphological characteristics of hot peppers were investigated. The hot peppers were grown under two CC scenarios, with the Representative Concentration Pathway (RCP) of 4.5 (Temp.; +3.4°C, CO_2_ conc.; 540 μmol/mol, Precipitation +17.3%) and RCP 8.5 (Temp.; +6.0°C and CO_2_ conc.; 940 μmol/mol, Precipitation +20.3%), respectively, using extreme weather simulators. This was compared with existing weather conditions occurring in Jeonju, South Korea in terms of air temperature, relative humidity, radiation, and precipitation. Overall, the plant height showed the highest under moderate CC conditions (RCP 4.5) among all the treatments tested. The number of leaves in the RCP 8.5 condition showed 7,739/plants, which was 2.2 times higher than that of the control. In addition, fruit shape was shortened and percentage dry matter was also the highest. The yield of hot pepper in the CC RCP 4.5 and 8.5 conditions were decreased by 21.5% and 89.2% when compared with that of the control, respectively. The days to harvest in the condition of CC scenarios were shortened from 5 to 13 compared with that of control, predominantly due to the increased air temperature. The results indicated that the severe RCP CC scenarios made reduction in the yields and negative affection on the fruit qualities. Overall, hot pepper was tolerant of mild CC scenarios of temperature × CO_2_ but was significantly affected by more extreme CC interacting parameter concentrations (or similar).

## 1 INTRODUCTION

Hot pepper (*Capsicum annuum* L.) is a key ingredient of Kimchi, considered to be an important health‐promoting food, both in South Korea and world‐wide. In South Korea, the cultivated area and production per year of hot pepper in 2015 were 34,514 ha and 97,000 tons, respectively. This was economically worth approx. 7.8 billion USD representing 2% of the country's total agricultural products (MAF Statistics, 2016). Hot pepper is predominantly cultivated in open field systems. The crop is thus exposed to the prevailing weather conditions in terms of temperature, radiation, and precipitation. It is thus vulnerable to extreme weather events. Previous studies have suggested that under high precipitation conditions, hot pepper is susceptible to outbreaks of anthracnose or phytophthora diseases which can significantly impact on yield (Hwang & Tae, 2001). Abnormal/extreme weather conditions in terms of extremely high temperature, heavy rainfall, and drought in summer due to climate change are known to be the main causes of a reduction in hot pepper production (Hwang, Kwon, Doh, & Park, 2010; Smittle, Dickens, & Stansell, 1994).

Climate Change (CC) models predict that the air temperature will rise by between 1 and 4°C by the end of the 21st century, because of increased greenhouse gases in the atmosphere (IPCC, 2007). The temperature of the earth increased by an average of 0.7°C during the past century, and the rate of temperature increase rate is increasing twice as rapidly in Korea since 1980 (IPCC, 2013). For this reason, the Korean Government has initiated research on mitigation and adaptation of key agricultural and horticultural crops to CC scenarios and to examine impacts of moderate and extreme CC scenarios by examining Representative Concentration Pathways (RCP) of 4.5 and 8.5 (NIMR, 2011). Air temperature is predicted to increase by approximately 6.0°C by 2100, the CO_2_ concentration is expected to increase to 940 μmol/mol, and the precipitation to increase by 20.4% from existing conditions under the RCP 8.5 CC scenario.

Previous research revealed that increases in air temperature (Heo et al., 2013, Lee, Lee, Kim, & Lee, 2001), CO_2_ concentration (Hansen, Sato, Ruedy, Lacis, & Oinas, 2000), and precipitation (Hwang & Tae, 2001) significantly retarded growth and reduced yield of hot pepper. Extreme hot summers lead to a decrease in the production of hot pepper by reducing both growth and increasing the occurrence of abnormal fruits (Heo et al., 2013; Lee et al., 2001). Sin and Yun (2010) founded that elevated CO_2_ and temperature increased the incidence of hot pepper diseases. Under intense rainfall conditions, the rhizosphere can become anaerobic, affecting root penetration and impacting on growth and plant photosynthesis (Lee, Park, Kim, Choi, & Lee, 2017). In addition, the amount of irrigation has been found to be closely correlated with size and weight of hot pepper fruits (Hwang et al., 2010; Sezen et al., 2014; Smittle et al., 1994). Physiological response studies with increasing air temperature and CO_2_ concentrations for cucumber (Taub, Seemann, & Coleman, 2000), radish, and Kimchi cabbage (Choi, Seo, Lee, Cho, & Stangoulis, 2011; Lee, Kim, Lee, Choi, & Park, 2016; Lee et al., 2009; Oh, Moon, Song, Son, & Koh, 2015) have shown retarded growth and deducted yield, as well as increased physiological disorders under extreme weather conditions. Studies have been carried out to develop prediction models of the effect of various environmental factors such as temperature and precipitation on crop growth and yield (Hadley et al., 1995; Poster & Semenov, 2005).

While several studies have examined the effect of single environmental factors on growth and yield of hot pepper, very few, if any, have examined the impact of interacting CC factors. The objective of this study was to compare the effect of existing growth conditions with moderate and extreme CC scenarios (RCP 4.5; 8.5) on (1) growth and development, (2) morphological characteristics, (3) photosynthesis, and (4) yield of hot pepper.

## 2 MATERIALS AND METHODS

### 2.1 Plant materials and agronomic practices

The plant materials were hot pepper plants (cv. “Supermanita,” Nongwoo Bio Co., Suwon, Korea) and seeds were sown on 16 February 2016 into a plug tray (35 ml/cell) filled with commercial soil (Bio media, Hungnong Seed Co., Korea) in a glasshouse at the National Institute of Horticultural and Herbal Science, located in Wanju (35°16′N, 127°02′E, 32 m above sea level). Air temperature was maintained at 25–30°C during the daytime and at 15°C at night time. Sixty‐four days after sowing, seedlings were transplanted into 2.7 m^3^ containers filled with a clay loam type of soil in the extreme weather growth chambers (EWGC; Modified CEEWS model, Environmental Growth Chambers, Chagrin Falls, OH, USA). The EWGC was equipped with artificial light system, a combination of 12 400‐W metal halide lamps (MS400, Philips Lighting, Amsterdam, Netherlands) and 12 400‐W high‐intensity‐discharge (HID) lamps (LU400, GE Lighting, East Cleveland, OH, USA). The plants were grown with 30 cm spacing in each row, at a plant density of 3.3 plants/m^2^. Plants were watered twice a day, and the following fertilizer program was applied in the experiment at planting (2,000 kg/ha for composts, 135.0 kg/ha for N, 64.0 kg/ha for P_2_O_5_, and 60.6 kg/ha for K_2_O). Additionally, the nutrient solution was applied approximately 1.5 L/plant weekly from 40 days after transplanting (DAT) to 171 DAT. The nutrient solution was composed of 808.8 mg/L KNO_3_, 944.6 mg/L Ca(NO_3_)_2_·4H_2_O, 153.4 mg/L NH_4_H_2_PO_4_, and 493.0 mg/L MgSO_4_·7H_2_O as macroelements. Microelement composition was 3 mg/L Fe as Fe‐EDTA, 0.5 mg/L H_3_BO_3_, 0.5 mg/L MnSO_4_·4H_2_O, 0.05 mg/L ZnSO_4_·7H_2_O, 0.02 mg/L CuSO_4_·5H_2_O, and 0.01 mg/L Na_2_MoO_4_·2H_2_O.

### 2.2 Climate change (CC) scenarios treatments

**Figure 1:**
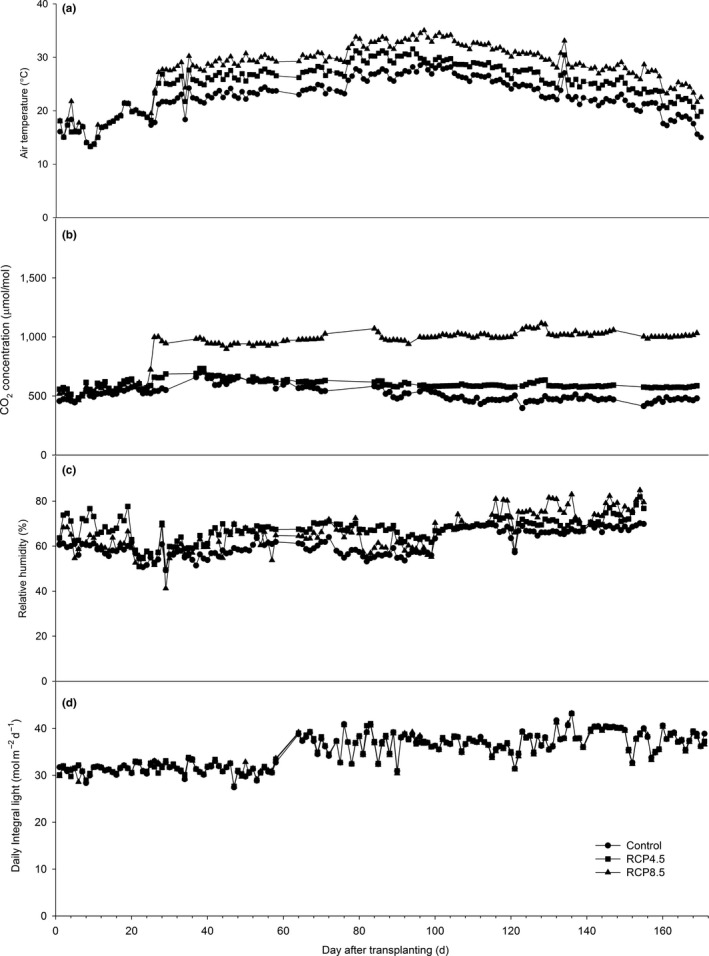
Changes in air temperature (a), CO_2_ concentration (b), relative humidity (c), and daily integral radiation (d) of each extreme weather simulators as applied by climate change scenarios

CC scenarios were adapted from the Representative Concentration Pathways (RCP) outlined in the Intergovernmental Panel on Climate Changes’ 4th Assessment Report (IPCC, 2007). The climate values were adjusted by the RCP scenario parameters and programed into the EWGCs using an advanced environmental control program (SIMATIC WinCC Runtime Advanced V13 SP1, Siemens, Munich, Germany) in terms of air temperature, CO_2_ concentration, and precipitation. The air temperatures for the RCP 4.5 and RCP 8.5 treatments were set at 3.4 and 6.0°C higher than the weather conditions based on the meteorological data from Jeonju region (three‐year mean; April to October 2013–2015) respectively, while their respective CO_2_ concentrations were maintained at 540 and 940 μmol/mol. In addition, precipitation of RCP 4.5 and 8.5 treatments was set at 17.3 and 20.4%, respectively more than that of the control. The control hot pepper treatment was transplanted and grown under the existing weather conditions in Jeonju, Korea. The mean air temperature of the control was 22.8°C during the experimental period, while that for the CC RCP 4.5 and 8.5 treatments was 25.2 and 28.1°C, respectively (Figure 1a). The CO_2_ concentration of the RCP 4.5 and 8.5 treatments represented 520 and 915 μmol/mol, respectively (Figure 1b). The mean relative humidity and daily integral light of all the tested treatments during experimental period were approx. 68% and 35 mol m^−2^ day^−1^, respectively (Figure 1c,d).

### 2.3 Measurements of growth of hot pepper plants and physical fruit characteristics

The plant height, leaf length and width, number of leaves, leaf area, and dry matter of hot peppers were estimated at 171 DAT. The three replicates of hot pepper plants were randomly selected in each treatment of the treatments. Plant height (cm/plant) was taken from the surface of soil to the top of the plant. The leaf area was measured using a leaf area meter (LI‐3100, LI‐COR Inc., Lincoln, NE, USA). In addition, the evaluation of chlorophyll content was assessed using a portable chlorophyll meter (SPAD 502, Minolta Camera Co., Ltd., Osaka, Japan). In addition, five fruits were sampled for physical traits in terms of length, width, fresh and dry weight records at 171 DAT.

### 2.4 Anatomical observation of hot pepper leaf structure

Leaf samples of the hot pepper were collected 170 DAT. The leaf samples were first fixed with 2.5% glutaraldehyde for 90 min at 4°C and then washed four to five times at 15‐min intervals in 0.1 mol/L phosphate buffer solution. The samples were subsequently fixed with 1% osmium tetroxide for 90 min, washed and then they immersed overnight. The samples were dehydrated through a series of graded ethanol concentrations (40, 60, 80, 90, and 95%) for 5 min and then with 100% ethanol for 5, 15, 15, and 30 min before being embedded in Epon (Electron Microscopy Sciences, Fort Washington, PA, USA) for 4 days at 60°C. The Epon blocks were cut into 1,500 nm sections with an ultramicrotome (Ultracut R, Leica Microsystems, North Ryde, Australia) and stained using periodic acid and Schiff's reagent, before being observed under a microscope (Axioskop 2, Carl Zeiss, Oberkochen, Germany) at 100 × magnification.

### 2.5 Measurements of photosynthetic characteristics of hot pepper plants

The CO_2_ assimilation rate (An) as a function of the intercellular CO_2_ concentration (Ci) curve (An‐Ci curve) and the A response to light intensity curve were measured from the leaves of 80 and 87 days old transplanted plants, respectively, using a portable photosynthesis system (Li 6400, LI‐COR Co., Inc., Lincoln, NE, USA). The relative humidity in the leaf chamber ranged from 60% to 80%, and the block temperature was maintained at 25°C. The gas exchange responses to CO_2_ concentrations ranged from 50 to 1,500 μmol/mol and measured at 500 μmol m^−2 ^s^−1^ photosynthetic photon flux (PPF), with a 10% ratio of blue light to maximize the stomatal aperture. Biochemical models (Farquhar et al., 1980) were used to determine the net photosynthetic rate, which is limited by the rates of CO_2_ carboxylation, by ribulose‐1,5‐bisphosphate regeneration, and by triose phosphate utilization (TPU). These limiting photosynthetic parameters were calculated for the hot pepper leaves using an Excel macro program developed by Sharkey, Bernacchi, Farquhar, and Singsaas (2007). For the light curve, the ambient CO_2_ concentration for the control, RCP 4.5 and RCP 8.5 plants were 400, 540, and 940 μmol/mol, respectively, and the same amount of CO_2_ was maintained in the leaf chamber during measurements. Measurements were taken at irradiance levels of 1,800, 1,500, 1,200, 1,000, 800, 500, 250, 100, 50, and 0 μmol m^−2 ^s^−1^ PPF, with three replicates performed per treatment. Regression analyses were performed for the calculation of the light saturation and compensation points using a descriptive, negative exponential model (Evans, Jakobsen, & Örgren, 1993).

### 2.6 Fruit production and days to harvesting of hot pepper

For evaluation of fruit production, the hot pepper fruits from nine replicate plants were collected during 84 DAT to 171 DAT. In addition, fruits were also harvested at the fully ripened stage. The ripened fruits were classified as normal and abnormal fruits based on calcium deficiency symptoms. The number of days required to harvest after flowering was measured in ten fruits from nine hot pepper plants at three different time periods (labeled flowering data at 47, 57, and 73 DAT, respectively).

### 2.7 Experimental designs and statistical analysis

A total of 18 hot pepper plants from nine replicates were grown in each of the three growth conditions, arranged in a completely randomized design. The different characteristics were statistically assessed by ANOVA in SAS (version 9.2, SAS Institute Inc., Cary, NC. USA). The means of the growth, physical fruit quality, and photosynthetic parameters were compared. Mean separation was analyzed using LSD tests, with a statistical difference recorded where *p* < .05.

## 3 RESULTS

### 3.1 Growth of hot pepper plant and physical fruit characteristics

The plant height, leaf length, number of leaves, chlorophyll content, and percentage of dry matter of hot pepper were significantly affected by CC scenarios (Table 1). The mean plant height in the RCP 4.5 treatment was 203 cm/plant, which was the highest of all the treatments. However, there were no significant differences between the control and the RCP 8.5 treatments. Under the extreme weather conditions (RCP 8.5), leaf length was decreased by 40.7% compared with the control, while number of leaves and percentage dry matter increased approx. 2.2 and 1.3 times, respectively. The chlorophyll content of leaves of the RCP 8.5 treatment was 59.6 SPAD value, which was the lowest among all the tested treatment. There was a significant difference for all the physical traits of hot pepper fruits between treatments (Table 2). The length and width of fruits of the RCP 8.5 treatment were significantly reduced by 22.3% and 62.5%, respectively. The width of fruits was more affected by CC exposure when compared with the effect on length of the fruit. In addition, fresh and dry fruit weights of the RCP 8.5 were the lowest at 4.1 and 0.8 g/fruit of all the treatments.

**Table 1:** Growth of hot pepper plants as affected by climate change scenarios at 171 DAT

Climate change scenarios	Plant height(cm/plant)	Leaf length (cm/plant)	Leaf width (cm/plant)	Number of leaves (/plant)	Leaf area (cm^2^/plant)	Chlorophyll content (SPAD value)	Percentage of dry matter (%)
Control	150b^a^	14.0a	5.8a	3,476b	21,353a	74.0a	19.6b
RCP 4.5	203a	15.3a	8.8a	4,509b	25,041a	67.4ab	20.0b
RCP 8.5	167b	8.3b	3.6a	7,739a	27,896a	59.6b	25.0a
LSD value	23.2	4.0	5.4	2,225	9,405	8.0	1.8

a Different letters within columns indicate significant differences by the LSD test at *p *=* *.05.

**Table 2:** Fruits traits of hot pepper as affected by climate change scenarios at 171 DAT

Climate change scenarios	Length (cm/fruit)	Width (cm/fruit)	Fresh weight (g/fruit)	Dry weight (g/fruit)
Control	10.3a^a^	19.5a	8.8a	1.5a
RCP 4.5	10.0ab	12.8b	6.4b	1.1b
RCP 8.5	8.0c	12.0c	4.1c	0.8c
LSD value	0.25	0.33	0.42	0.07

a Different letters within columns indicate significant differences by the LSD test at *p *=* *.05.

### 3.2 Morphological features of hot pepper leaf structure

**Figure 2:**
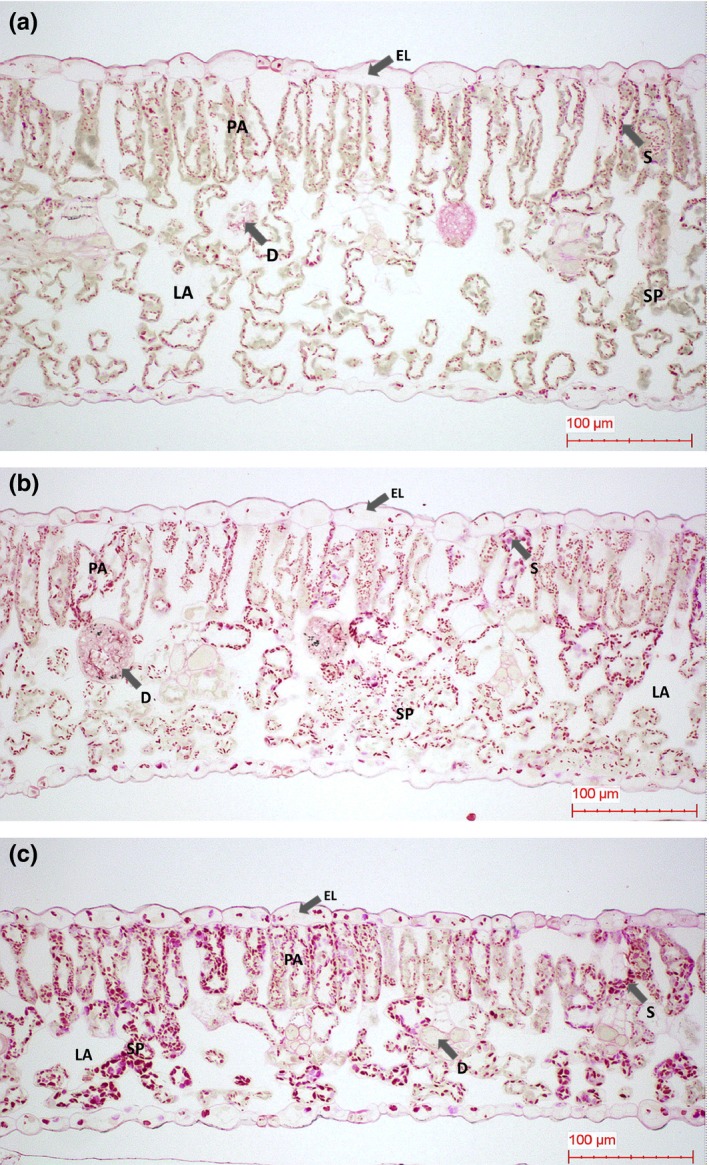
Cross‐sections of hot pepper leaves with control (a), RCP 4.5 (b), and RCP8.5 (c). EL, adaxial epidermal layer; PA, palisade; SP, spongy parenchyma; D, druse crystals; LA, lacunae in spongy mesophyll tissues; S, starch

Cross‐sections of hot pepper leaves indicated that the high temperature/elevated CO_2_ concentration (RCP 8.5) CC scenario induced structural modifications (Figure 2a–c). Hot pepper leaf thickness was similar in all the tested treatments. However, the density of epidermal cells was higher than in control leaves. Intercellular air spaces were significantly reduced by both CC treatments and the number of both palisade and spongy mesophyll parenchyma cells were more apparent in these CC treatments. In addition, in the moderate RCP 4.5 CC treatment leaves, there was a more marked tendency for the development of two layers of palisade parenchyma. In the more extreme CC scenario (RCP 8.5) leaves, more starch grains and a stronger coloration compared to the control treatment.

### 3.3 Photosynthetic characteristics of hot pepper plants

**Figure 3:**
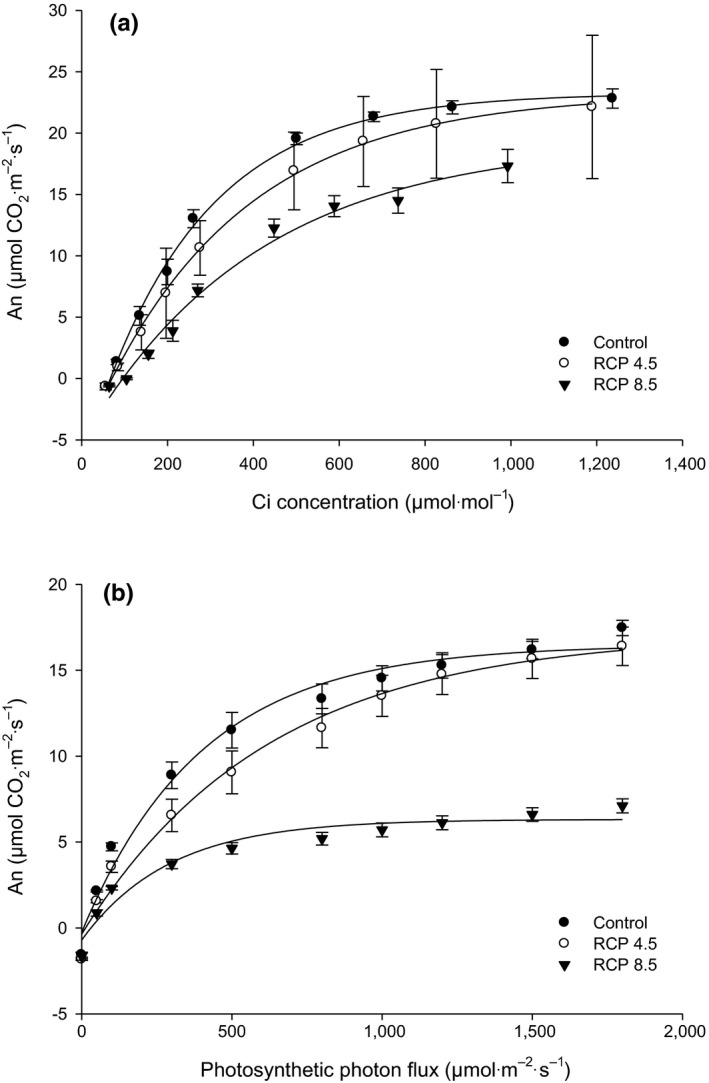
An‐Ci (a) and light curves (b) of hot pepper leaves as affected by climate change scenarios at 80 and 87 days after transplanting, respectively

The maximum CO_2_ assimilation in the RCP 8.5 showed 17.3 μmol CO_2_ m^−2 ^s^−1^. This was 24.1% lower than that of control in the An‐Ci response curve. However, there was no significant difference between the control and the moderate RCP 4.5 treatments (Figure 3a). In addition, the CO_2_ compensation point of RCP 8.5 treatment was approx. 97 μmol/mol CO_2_ concentration, which was significantly higher than the other treatments (data not shown). In the light curve, the maximum CO_2_ assimilation of the RCP 8.5 treatment was significantly reduced by 56.7%–59.4% when compared with the other treatments. Indeed, the light saturation point of the RCP 8.5 treatment represented 300 μmol m^−2 ^s^−1^ PPF, which was the lowest among all the tested treatments (Figure 3b). The light compensation point of control was 7.0 μmol m^−2 ^s^−1^ PPF, while that of RCP 4.5 and 8.5 treatments was 17.5 and 16.7 μmol m^−2 ^s^−1^ PPF, respectively. These were significantly higher (2.4 times) than that of the control (data not shown). The assessment of photosynthetic efficiency using the An‐Ci curves was conducted using the Farquhar‐von Caemmerer‐Berry's biochemical model (FvCB model). The results indicated that the maximum carboxylation rate, maximum rate of electron transport, triose phosphate utilization rate, and mitochondrial respiration in light of leaves treated with RCP 8.5 were 37.9, 41.4, 5.2, and 1.6 μmol m^−2 ^s^−1^, respectively, which were significantly lower than those of the control (Table 3).

**Table 3:** Estimated photosynthetic parameters of the biochemical model for hot pepper at a leaf temperature of 25.0°C

Climate change scenarios	V_max_ a (μmol m^−2 ^s^−1^)	J_max_ b (μmol m^−2 ^s^−1^)	TPU^c^ (μmol m^−2 ^s^−1^)	R_d_ d (μmol m^−2 ^s^−1^)
Control	71.1a^e^	84.7a	7.5a	2.4a
RCP 4.5	57.9b	68.3b	6.7ab	1.6b
RCP 8.5	37.9c	41.4c	5.2b	1.6b
LSD value	12.0	15.8	1.6	0.4

a Maximum carboxylation rate.

b Maximum rate of electron transport.

c Triose phosphate utilization rate.

d Mitochondrial respiration in the light.

e Different letters within columns indicate significant differences by the LSD test at *p *=* *.05.

### 3.4 Fruit production and days to harvesting of hot pepper

**Figure 4:**
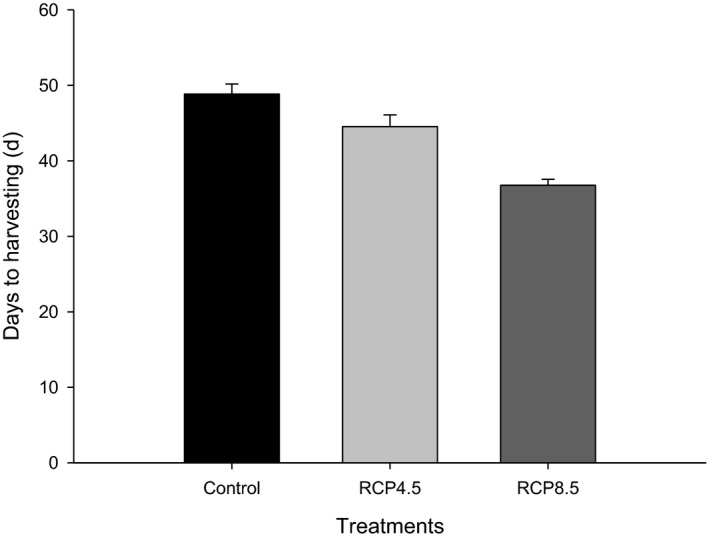
The days to harvesting of hot pepper as affected by climate change scenarios

The total number of fruits in the control, RCP 4.5 and RCP 8.5 treatments from nine replicate plants was 210, 249, and 59/plant in the harvest period from 84 to 171 DAT. This shows that in the RCP 8.5 treatment, the fruit number was significantly reduced (Table 4). The mean fresh weight of control showed 6.30 g/fruit, which was greatest among all the tested treatments. The yield in the RCP 8.5 treatment decreased by 89.2% compared with the control. In addition, the production of abnormal fruits in terms of calcium deficiency symptoms occurred more often in the moderate CC scenario (RCP 4.5). The number of days to harvesting of the control was approximately 49 days, while that for the RCP 8.5 treatment was 37 days (Figure 4). Extreme higher temperatures and increased CO_2_ concentration thus reduced the ripening periods for hot pepper fruits.

**Table 4:** Fruit production of hot pepper plants as affected by climate change scenarios during approximately 3 months, from 84 to 171 DAT

Climate change scenarios	Normality fruits				Number of abnormality fruits (/plant)
	Number of fruits (/plant)	Fresh weight (g/fruits)	Yield (kg/10a)^a^	Index	
Control	210ab^b^	6.3a	4,405	100	12c
RCP 4.5	249a	4.2a	3,457	78.5	28a
RCP 8.5	59b	2.4b	477	10.8	18b
LSD value	152.2	2.29	–	–	4.3

a The yield was calculated as number of fruits in normality fruits × fresh weight × plant density (3,330 plants/10a)/1,000.

b Different letters within columns indicate significant differences by the LSD test at *p *=* *.05.

## 4 DISCUSSION

### 4.1 Growth of hot pepper plant and physical fruit characteristics

**Figure 5:**
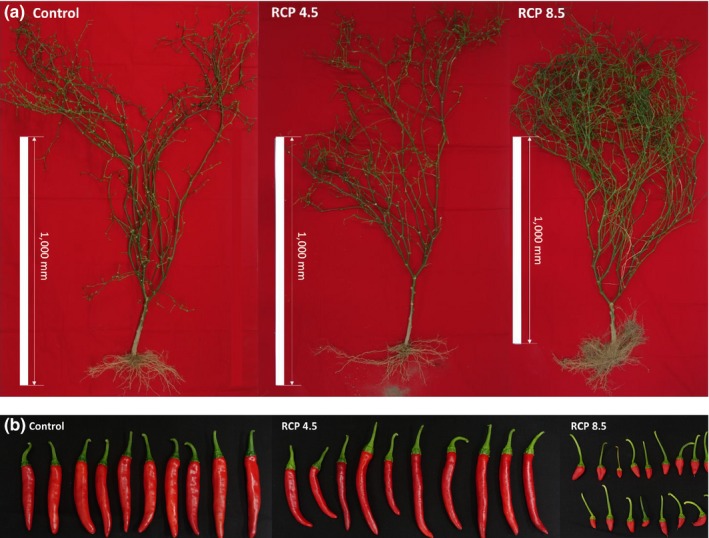
The morphological characteristics of plants (a) and fruits (b) of hot pepper as affected by climate change scenarios. The bars of b represent 50 mm

The CC factors of elevated air temperature and CO_2_ concentrations affected both growth and fruit development of hot peppers. For optimal growth, experts recommend that night air temperature should be not more than 30°C in hot pepper (Dorland & Went, 1947). However, daily air temperature in the RCP 8.5 treatment during the experiment period was approximately 30°C (Figure 1a), thus, leaf morphogenesis was abnormal, with the rate of leaf enlargement and appearance affected. In addition, N uptake was reduced and the degradation rate of chlorophyll was enhanced by the CC treatments. For example, the RCP 8.5 treatment leaves were slightly yellow in appearance (59.6 SPAD; Table 1). Previous results have suggested that chlorophyll in Kimchi cabbage leaves is also reduced by CC exposure (Lee et al., 2016). The hot pepper plant normally grows with a single stem, until 9–11 leaves have been formed. The main stem terminates with a flower and then normally two branches grow from the axils of the highest leaves. Figure 5a shows that two branches were induced in the control hot pepper plants. However, in the RCP 4.5 and 8.5 treatments, three or more branches were induced. This suggests that vegetative growth may be stimulated when exposed to such CC scenarios. Also, pollination and fertilization of hot pepper are significantly decreased at >30°C air temperature conditions (Erickson & Markhart, 2002; Khah & Passam, 1992; Pagamas & Nawata, 2008). Also, at <35°C night air temperature condition, the pollen viability and pollen tube behavior are reduced (Gajanayake, Trader, Reddy, & Harkess, 2011; Usman, Mamat, Mohd, Aishah, & Annuar, 1999). Thus, the control and RCP 4.5 treatments had normal shaped hot pepper fruits, while the RCP 8.5 treatment showed abnormal development (Figure 5b). The relative humidity was not varied among treatments; the control, RCP 4.5, and RCP 8.5 were similar (68%) during experiment treatment. Therefore, precipitation was not more effects on growth and physical fruit characteristics of hot pepper than air temperature and CO_2_ conditions.

### 4.2 Morphological features of hot pepper leaf structure

Previous studies by Downton and Tregunna (1968) found well‐developed parenchyma bundle sheaths containing a high concentration of chloroplasts which accumulated large amounts of starch in CO_2_ compensation plants. This sink/source relationship may influence the fruiting development, and in the CC RCP 8.5 treatment, they were smaller than in the control. This suggested that the sink strength was relatively lower. In addition, elevated CO_2_ concentration increased the net CO_2_ assimilation. However, this induced physiological disorders of fruits. Lee et al. (2016) revealed that Kimchi cabbage exposed to RCP 8.5 CC scenario had unformed heads and abnormal development of mesophyll cells.

### 4.3 Photosynthetic characteristics of hot pepper plants

The net photosynthetic rate of hot pepper leaves declined in air temperatures >35°C. This may be partially attributable to higher rates of respiration at increased temperatures. The FvCB model is a predictor in the assessment of plant responses to CO_2_, which was used to simulate photosynthesis under changing environments. The FvCB model has been used in a wide range of physiological studies on crop plants, from estimating the underlying C3 leaf biochemistry to predicting the photosynthetic fluxes of ecosystems in response to climate change (Yin & Struik, 2009). Previous long‐term studies on Kimchi cabbage exposed to elevated CO_2_ showed a greater influence on photosynthesis (Choi et al., 2011; Lee et al., 2016), decreasing the net photosynthetic rate, stomatal conductance, and internal CO_2_ concentration. Choi et al. (2011) suggested that this might occur because decreases in photosynthesis at elevated CO_2_ concentrations are associated with a decreased demand for carbohydrates. The information from hot pepper using the An‐Ci curve might help to better understand these physiological responses in conditions where CO_2_ concentration, light intensity, and air temperature can accurately be controlled. Therefore, results of photosynthesis indicated that RCP 8.5 CC conditions severely negatively impacts on net photosynthetic rate and photosynthesis efficiency of currently common cultivars of hot pepper.

### 4.4 Fruit production and days to harvesting of hot pepper

Previously, high air temperatures caused significant losses in productivity of many crop species due to reduced seeds and increased flower abscission or abortion (Konsens, Ofir, & Kigel, 1991; Wheeler, Craufurd, Ellis, Porter, & Vara Prasad, 2000). The yield of hot pepper has been shown to be increased at 2°C higher than optimal growth temperature. However, in extreme high temperatures, this decreased (Song et al., 2015). Heo et al. (2013) founded that exposure of hot pepper during early growth to high temperature and CO_2_ concentrations (until 90 days after sowing) increased the growth rate. However, the yield was significantly reduced. In addition, based on the parameter of days to harvest, in the RCP 8.5 CC treatment, the heat unit was shortened by 24.5% in hot pepper.

## 5 CONCLUSION

This study found that the severe RCP CC scenarios reduced growth and yields of hot pepper. There were also negative effects on morphogenesis, photosynthesis, and fruit characteristics. However, in the moderate CC RCP 4.5 scenario, growth of hot pepper was more resilient with minimum effects on growth and yield. This suggests that the future breeding programs should focus on practical resilience of hot pepper cultivars to be able to cope with extreme CC scenarios. This will be important to ensure that a supply of this key ingredient for Kimchi in South Korea is not affected by predicted CC events.

## CONFLICT OF INTEREST

The authors declare that they have no competing interests.

## AUTHOR'S CONTRIBUTIONS

SGL and SKK initiated and designed the experiments. SKK analyzed the data and wrote the manuscript. HJL, HSL, and JHL carried out the experiments. All authors read and approved the manuscript.
